# The Fontan pathway: What's down the road?

**DOI:** 10.4103/0974-2069.43872

**Published:** 2008

**Authors:** Sachin Khambadkone

**Affiliations:** Great Ormond Street Hospital and Institute of Child Health, London, United Kingdom

**Keywords:** Failing Fontan, protein losing enteropathy, cardiac transplantation

## Abstract

The Fontan circulation results from routing of the systemic venous blood to the pulmonary circulation without a hydraulic source of a ventricle. Although a hypertrophied right atrium was thought to be essential for this circulation, the current form of the operation has neither the right atrium nor any valves in the venous circulation that is connected to the pulmonary arteries directly. Modifications in the operative model was one of the early steps in improving outcome. Use of fenestration, staging of Fontan completion and better perioperative management have led to a significant drop in mortality rates in the current era. Despite this, there is late attrition of patients with complications such as arrhythmias, ventricular dysfunction, and unusual clinical syndromes of protein-losing enteropathy (PLE) and plastic bronchitis. Management of failing Fontan includes a detailed hemodynamic and imaging assessment to treat any correctable lesions such as obstruction within the Fontan circuit, early control of arrhythmia and maintenance of sinus rhythm, symptomatic treatment for PLE and plastic bronchitis, manipulation of systemic and pulmonary vascular resistance, and Fontan conversion of less favorable atriopulmonary connection to extra-cardiac total cavopulmonary connection with arrythmia surgery. Cardiac transplantation remains the only successful definitive palliation in the failing Fontan patients.

## INTRODUCTION

We have entered the fourth decade after the first clinical report by Fontan and Baudet, of an operation for ‘surgical repair of tricuspid atresia’.[1]

**Figure 1:**
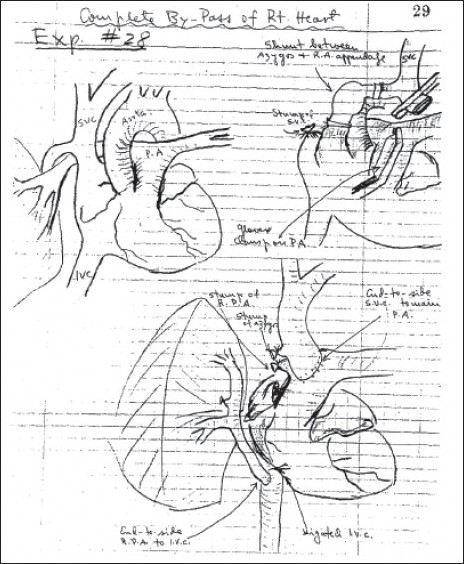
The original description of bidirectional Glenn as a temporizing measure in total right heart bypass experiments. Reproduction of Dr. Jose Patino's diagrams from the protocol books. Reprinted from The Journal of Thoracic and Cardiovascular surgery, vol 114, issue 6, Glenn WWL; A temporary bidirectional superior vena cavapulmonary artery shunt; page 1124, 1997, with permission from Elsevier

**Figure 2:**
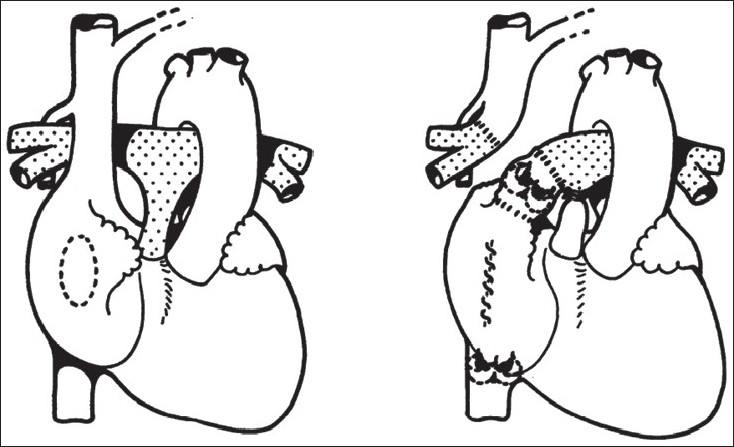
The depiction of the Fontan operation in Dr. Francis Fontan's original description in Thorax 1971. Fontan F and Baudet E, Thorax 1971, Vol 26/ Issue 3. 240 – 248: adapted and reproduced with permission from the BMJ Publishing Group

Although the title implied a definitive and corrective operation, the basic principles of right heart bypass were expanded from the early reports in animal experiments by Rodbard and Wagner[2], and by Glenn and Patino.[3] Glenn also attempted total right heart bypass where a bidirectional superior cavopulmonary connection was described with inferior caval anastomosis to right pulmonary artery[4] [Figures 1 and 2]. In fact, many groups worked simultaneously on variants of right heart bypass in the 1950s.

Many people have quoted and referenced the original report by Fontan and Baudet, but few may realize some of the concepts tried by the pioneers of the Fontan circulation.

**Figure 3:**
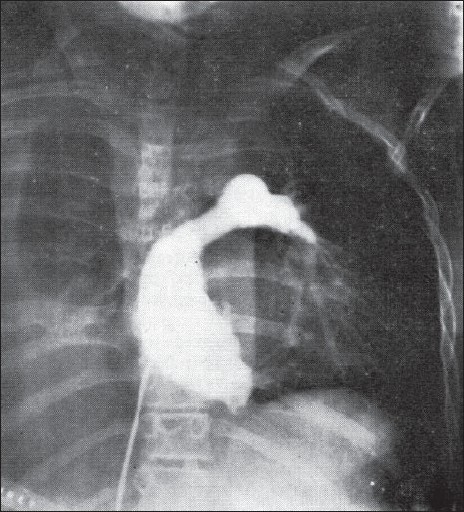
The angiogram in Fontan's fist patient showing filling of the left pulmonary artery. The right pulmonary artery was anastomosed to the upper part of right atrium akin to the Classical Glenn operation. Fontan F and Baudet E, Thorax 1971, Vol 26/ Issue 3.240–248: adapted and reproduced with permission from the BMJ Publishing Group

**Figure 4:**
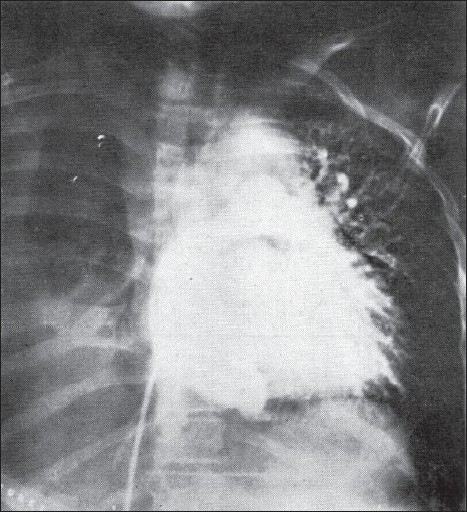
The role of a valve at the inferior vena cava-right atrium junction to prevent retrograde flow in the inferior vena cava in the first Fontan operation. Fontan F and Baudet E, Thorax 1971, Vol 26/ Issue 3. 240–248: adapted and reproduced with permission from the BMJ Publishing Group

All patients in the first series had tricuspid atresia, as they believed that ‘ventricularization’ of a hypertrophied right atrium in this morphological substrate would be adequate to support the pulmonary circulation. The atrium in the atriopulmonary connection was, in fact, thought to be a worthy substitute for a dimunitive right ventricle. In the first report,[5] of the three cases, the first case had a direct connection of the right pulmonary artery to the right atrial appendage, however, the next two had an aortic homograft placed as the atriopulmonary conduit. Furthermore, all three patients had a pulmonary homograft in the inferior caval position [Figures 3 and 4]. In fact, valvulation in the Fontan circuit was thus, attempted right from the first clinical description of this circulation and is not a new concept.[6]

There are a few other pearls of wisdom from the pioneering report. The need for ‘large fluid infusions’ and to ‘maintain tachycardia’ for ‘correct hemodynamic balance’ was recognized in the early postoperative period. Detrimental effect of positive-pressure ventilation on the venous return was also indentified and early extubation recommended. The unpredictable hemodynamics resulting from atrial arrhthymias such as flutter or fibrillation was also recognized.

In one sentence, the authors encompass the criteria for suitability thus, “patients were anatomically and haemodynamically privileged; they had pulmonary arteries of normal size and low pressure”.

The basic principles of right heart bypass opened up a novel option for patients with a single dominant ventricle, be it left or right, and for patients with intracardiac mixing where septation of the atria or ventricles could not be achieved.

The Fontan circulation, rather than the ‘Fontan operation’, thus, became the final step in surgical treatment of single dominant ventricle circulation.

### The final operation

Early evolution of the Fontan operation involved modifications in achieving the cavopulmonary connection. The premise that a pulsatile chamber, namely the right atrium, is essential to propel blood into the pulmonary circulation was questioned. In a series of elegant experiments, de Leval and colleagues elucidated the detrimental effects of a pulsatile valveless right atrium in the cavopulmonary circuit. Instead of providing power, it contributed to turbulence, energy loss, and increased resistance to net forward flow. They proposed a hydrodynamically more efficient total cavopulmonary connection[7] that provided some advantages – technically simple, reproducible in any atrioventricular (AV) arrangement, away from the AV node, a low pressure right atrium with reduced risk of early or late arrhythmias, reduced turbulence, less energy losses, and lower risk of atrial thrombosis. This was achieved either with creation of a lateral tunnel or placement of an intratrial conduit.

The use of extracardiac conduits of biological or prosthetic tubes was the next step to exclude the right atrium from the venous rerouting. The total cavopulmonary connection is generally achieved with a low mortality in most centers. As the long-term complications of atriopulmonary connection were obvious during the late 1980s and early 1990s, the total cavopulmonary connection by various techniques was generally accepted as the final design of the Fontan circulation.[8–13]

The introduction of fenestration of the Fontan circuit[14–16] created a significant impact on early morbidity and mortality. It reduced the incidence of postoperative pleural effusions and reduced the hospital stay. However, over the last few years, with meticulous patient preparation and selection, routine fenestration during Fontan completion has no longer been necessary in patients with good hemodynamics.[17–21]

The staging of cavopulmonary connection with introduction of an intermediary step of a hemi-Fontan or the bidirectional Glenn operation (superior cavopulmonary anastomosis) with its advantage of early volume reduction, further improved results and expanded the case selection to include some of the high-risk patients, by the then conventional indications.[22–24]

Although first described for tricuspid atresia, the Fontan circulation has been applied to a wide spectrum of complex congenital heart disease with a dominant single ventricle. Improvement in surgical techniques, improved understanding of perioperative physiology, and better selection and modification of the high-risk factors led to better results. In the early era of Fontan operation, mortality in large reported series was between 17–31%,[25] compared to 4.5–7% reported in the more recent series in the 1990s.[26,27]

During this period of change, apart from the operative techniques and perioperative management, the timing of intervention and emphasis on Fontan ‘preparation’ have contributed significantly to this improvement in outcome. In an interesting single institutional analysis, there was no difference in the risk factors and patient characteristics between a higher and lower mortality-risk era, indicating that differing interventions and changing treatment algorithms along with some other factors such as use of modified ultrafiltration after bypass and the use of extracardiac conduits contributed to the improvement.[28]

More recently, the National Heart Lung and Blood Institute funded Pediatric Heart Network designed a cross-sectional study of children aged 6–18 years, from seven pediatric clinical centers, who had undergone a Fontan procedure as treatment for congenital heart disease.[29] The aim was to record the characteristics of contemporary Fontan survivors with measures of ventricular systolic function, functional health status determined by child health questionnaire, and exercise performance.[30] The study found that ventricular systolic function and functional health, although lower than average, was within two standard deviations from the mean for control group. The cohort had impaired exercise performance with the mean percent of predicted peak VO2 being 65%. Again, the poor outcome in patients with morphological right ventricle was obvious, highlighting the need to optimize ventricular loading conditions before and during long-term follow-up.

### What leads to Fontan failure?

It should not have been a surprise when Fontan and colleagues warned of late attrition after what was deemed to be a ‘perfect’ Fontan circulation.[31] The essence of the Fontan circulation is in its imperfection levied by the morphological substrate.

Various attempts have been made to delay the onset of failing Fontan circulation based on the pathophysiology of clinical syndromes that manifest in its failure.

### The systemic veins

The superior and inferior caval veins are exposed to a higher pressure that generates the energy for pulmonary perfusion in absence of a hydraulic force, namely, the right ventricle. The Fontan circulation introduces a paradox of systemic venous hypertension (mean pressure > 10 mm Hg) and pulmonary arterial hypotension (mean pressure < 15 mm Hg), a step-down in the pressure as opposed to the normal step-up introduced by interposition of right ventricle, in a normal biventricular circulation.[32]

Systemic venous hypertension has detrimental effects on the infradiaphragmatic venous circulation, and more importantly the splanchnic circulation, due to the additional negative effect of gravity. There is loss of the hepatic venous pressure gradient due to passive distension and ‘open tube’ phenomenon, and the pressure is transmitted to the portal venous system leading to portal venous hypertension.[33,34]

The splanchnic venous hypertension contributes to protein-losing enteropathy (PLE), albeit, not being its sole cause. Altered flow in the gut vasculature, low cardiac output state, persistent hypoxia, and inflammation are the other contributors.[35]

### The systemic ventricle

The dominant systemic ventricle may be of right ventricular or indeterminate morphology with unfavorable fiber arrangement that is mechanically inefficient to sustain systemic cardiac output. On this morphological substrate, additional loading insults before establishment of Fontan circulation predispose the systemic ventricle to fail. Volume overload related to systemic to pulmonary artery shunt or pulmonary artery band that is obligatory after early palliation, valvular regurgitation from the systemic atrioventricular or semi-lunar valves, or pressure overload related to suboptimal systemic outflow tract hemodynamics (restrictive ventricular septal defect in discordant ventriculoarterial connection or recoarctation after hypoplastic left heart syndrome surgery) all act as early determinants of the failing ventricle.

From a volume overloaded, dilated, and hypertrophied ventricle, the superior cavopulmonary connection and finally the Fontan completion expose the systemic ventricle to volume unloading at suboptimal preloads. Following total right heart bypass, the preload of the systemic ventricle is reduced to 25–70% with respect to the ventricular size. This leads to systolic and diastolic dysfunction and myocardial dys-synchrony.[36] Chronic preload depletion perpetuates the diastolic dysfunction with impaired compliance, remodelling, and eventually low cardiac output.[37]

### The pulmonary circulation

Pulmonary vascular resistance (PVR) becomes an important determinant of cardiac output in the Fontan circulation where the systemic circulation is in series with the pulmonary circulation without an intervening hydraulic source of energy (viz. right ventricle).[38]

The pulmonary arteries may be morphologically abnormal, small, discontinuous, stenosed, or have abnormal arborization. The branch pulmonary arteries may be distorted during restriction of blood flow (e.g., migrated pulmonary artery band) or augmentation of flow (e.g., related to shunt). Ductal tissue could lead to coarctation of the branch pulmonary artery.

The pulmonary venous drainage, if anomalous, particularly with isomerism, has a higher potential of being obstructive leading to altered pulmonary arteriolar growth and potentially elevated pulmonary arteriolar resistance.

Even with morphologically ‘normal’ pulmonary arteries, a dominant single ventricle circulation exposes the pulmonary vasculature to different periods of too much or too little flow. The absence of pulsatile flow and a low pulmonary artery mean pressure underfills the pulmonary vascular bed and effectively increases PVR. Pulsatile flow is important for shear-stress-mediated release of endothelium-derived nitric oxide (NO) and for recruitment of pulmonary capillaries and to maintain their patency. The systolic pressure rise not only recruits the capillary bed and lengthens the capillaries but also keeps them open during diastole. After total cavopulmonary connection, there is loss of pulsatility in the pulmonary arterioles and capillaries with less recruitment of the pulmonary vascular bed.[39] A chronically underfilled pulmonary vascular bed with loss of pulsatility may contribute to pulmonary endothelial dysfunction and consequently increase pulmonary vascular resistance.[40]

Stasis, polycythemia due to chronic cyanosis, and abnormal coagulation profile in patients with Fontan circulation predispose them to increased incidence of pulmonary thromboembolism. Silent thromboembolic episodes have been documented in adults with Fontan circulation during late follow-up. However, there is no clear evidence that anticoagulation prevents thromboembolism in failing Fontan, or that in a hemodynamically stable circulation, routine anticoagulation is necessary to prevent morbidity and mortality.[41]

Collateral circulation through bronchial and other vessels remains a hidden unaccounted source of pulmonary blood flow that is difficult to quantify.[32] Although better preparation in the recent era has reduced the potential for extensive arterial collateralization, venous channels between systemic and pulmonary veins or hepatic veins and atrium still contribute to mixing. Assessment of pulmonary blood flow is incomplete without including the collateral flow in accurate estimation of pulmonary vascular resistance.

### Protein-losing enteropathy

Historically, chronic passive congestion in the systemic venous circuit, such as in chronic heart failure or constrictive pericarditis has manifested in lymphatic dysfunction with pleural or peritoneal leaks or leak from the gastrointestinal system.

High systemic venous pressure is the essence of the Fontan paradox where a higher than normal systemic venous pressure is necessary to maintain pulmonary blood flow. In failing Fontan, this systemic venous hypertension is transmitted to the venous and the lymphatic circulation. The true impact on the lymphatic system has not been fully understood due to the difficulties in imaging lymphatics.

PLE is an unusual manifestation of failing Fontan circulation with an unknown etiology. Its true prevalence may never be known as the protein loss from gastrointestinal tract probably starts at subclinical levels well before hypoalbuminemia and fluid accumulation manifests overtly. Elevated systemic venous pressure may increase the risk; however, is by no means the only cause of PLE.[42] Hence, it is not uncommon to have ‘normal’ Fontan pressure during cardiac catheterization. However, this finding may be flawed due to the low intravascular volume and low cardiac output state invariably associated with florid PLE, often compounded by treatment with diuretics. Peripheral edema, ascites, diarrhea, pleural effusions, or chylothoraces may lead to hypoproteinemia, immunodeficiency from loss of immunoglobulins and lymphocytes, and coagulopathy. A low cardiac output state may be an important contributory factor.[43] Interventions to increase the cardiac output such as fenestration in the Fontan circuit,[44,45] pacing,[46] or cardiac transplantation[1,47,48] may help in reducing the systemic venous pressure. The improvement in PLE with anti-inflammatory medications such as steroids and unfractionated heparin that has a membrane stabilizing effect imply that inflammation may contribute to the onset or continuation of this process.[49–53]

Abnormal flow distribution imposed by the low cardiac output state may cause impaired mesenteric flow contributing to development of PLE.[54] Along with splanchnic venous congestion, it may affect the intestinal mucosal cell function and induce apoptosis with subsequent loss of integrity and protein leakage.[55,56]

Low flow in chronic congestive heart failure is known to activate the cascade of inflammatory mediators(57) including cytokines such as tumor necrosis factor alpha (TNF-α). A similar pattern is seen in the failing Fontan circulation.[58] The TNF-α has been known to increase protein loss from the gastrointestinal tract by compromising the epithelial tight junctions. If heparan sulfate deficiency combines with increased levels of TNF-α, there is a significant synergistic effect on the albumin loss from the gut, hence, the use of heparin may be effective in PLE.[59]

Plastic bronchitis is an even rarer complication of Fontan failure. It manifests with chronic respiratory symptoms such as persistent cough, wheezing, and expectoration of bronchial casts. Although no clear etiological factors have been identified, elevated systemic venous pressure and elevated PVR are associated findings. Lymphatic dysfunction is also suspected, akin to that in PLE. The reason why some patients manifest with plastic bronchitis and others with PLE is not clearly understood. However, patients with Fontan circulation exposed to high altitude have been described to manifest both of these unusual symptom complexes.[60,61]

### Liver in failing Fontan

The elevated systemic venous pressure has an important effect on the subdiaphragmatic venous hemodynamics that includes the splanchnic and the hepatoportal venous system.[33] Chronic congestive heart failure leads to increased sinusoidal pressure and hepatic dysfunction, and in severe cases, cardiac cirrhosis. The earliest report of liver pathology was in a Fontan patient undergoing cholecystectomy. The liver biopsy showed severe fibrosis that was attributed to the chronic systemic venous hypertension.[62] Postmortem pathological examination of patients dying after Fontan circulation has shown different stages from chronic passive congestion to hepatocellular carcinoma.[63]

Biochemical liver dysfunction is common during late follow-up of Fontan patients, with elevation of transaminases, coagulation abnormalities, and elevation of bilirubin.[64–66] More recently, Kiesewetter and colleagues reported hepatic fibrosis and cirrhosis diagnosed by computed tomography of the liver that correlated with elevated hepatic venous pressure and long duration of the Fontan state.[67] Kendall and colleagues describe histopathological changes of sinusoidal dilatation and fibrosis in patients who were subjected to a liver biopsy as a part of preoperative investigations before Fontan conversion. Of the 18 patients, two had established cirrhosis and almost all had some grade of fibrosis with presence of orcein, indicating chronicity and irreversibility. There was no evidence of inflammation indicating a hemodynamic basis to these changes.[68]

The detrimental effects of gravity and abnormal respiratory function in patients with diaphragm paralysis may accelerate negative effects on the liver.[69]

### Arrhythmias

Hemodynamic decompensation due to loss of sinus rhythm was identified by Fontan in his first report of three cases. Subsequently, the atriopulmonary connection was recognized as the basis of atrial re-entry and atrial flutter as a consequence of dilatation, hypertension, and atrial scarring.

The early enthusiasm in the hydrodynamic benefit with lateral tunnel Fontan was tempered by lack of improvement in the early or late onset arrhythmias.[70] The extracardiac Fontan appears promising in terms of lowering the incidence of late onset arrhythmia by total exclusion of the right atrium.[71,72] This shows the improvement with changing strategies of Fontan operation; however, some other risk factors related to isomerism, atrioventricular valve regurgitation, and ventricular dysfunction may be difficult to eradicate.

Thus, control of arrhythmia is important to maintain good hemodynamics, prevent thromboembolism, and ventricular dysfunction.

### Treatment modalities for failing Fontan circulation

The earliest sign of failing Fontan circulation is a progressive deterioration in the absence of a clear precipitating cause, such as stenosis in the Fontan circuit. Every patient with suspected failure should have a detailed hemodynamic assessment of the Fontan circulation including meticulous imaging. Any correctable causes should be promptly treated, such as – stenting of branch pulmonary arteries, occlusion of systemic to pulmonary artery collaterals and occlusion of venous collaterals. Maintenance of sinus rhythm is paramount.

### Medical interventions directed at the systemic ventricle

Assessment of systolic and diastolic function of single ventricle is difficult and treatment modalities are usually extrapolated albeit wrongly from heart failure in biventricular circulation.[73] Betablockers have been anecdotally shown to improve hemodynamics in failing Fontan circulation.[74] The manipulation of systemic and pulmonary vascular resistances in Fontan has a directly interconnected effect on each other, different from that seen in biventricular circulation. Cardiac resynchronization therapy has also been attempted in Fontan ventricles with short-term improvement of hemodynamics and symptoms.[75]

### Pulmonary vasodilators in Fontan

The impact of vasodilators on early pulmonary vascular dysfunction was clearly demonstrated by Goldman *et al*, in postoperative period after Fontan operation.[76] Effect of exogenous NO on measured PVR was demonstrated by Khambadkone *et al*, in a cohort of Fontan patients during late follow-up.[40] With the advent of pulmonary vasoactive drugs such as phopshodiesterase inhibitors (sildenafil) and endothelin antagonists (bosentan), manipulation of PVR has been attempted in both early and late postoperative phases in Fontan circulation. They have been used to treat late sequelae such as PLE and plastic bronchitis, wherein elevated PVR and consequently, increased systemic venous pressure may have contributory effects.[77,78] Sildenafil has also been shown to acutely improve exercise performance and hemodynamic response to exercise.[79] Despite this, the long-term effect of sildenafil or bosentan on Fontan circulation and their tolerance is not known.[80]

### Ventricular assist in Fontan circulation

The advances in treatment of heart failure have been applied to the failing single ventricle circulation.[81] Initial application of ventricular assist was to bridge a failing systemic ventricle leading to Fontan failure to cardiac transplantation early after Fontan completion.[82,83] Use of ventricular assist for high PVR was recently demonstrated as a bridge, in presence of good systemic ventricular function.[84] The patient had a good relief of symptoms of high systemic venous pressure and ascites and had a successful cardiac transplant one year later.

### Fontan conversion

The evolution of Fontan operation has made the atriopulmonary Fontan connection obsolete as a procedure, however, the late survivors of this operation have provided a better understanding of complications. Progressive dilatation of the right atrium, atrial arrhythmia, thromboembolism, and exercise intolerance are the late consequences that lead to evaluation of these patients for reinterventions. Removal of the dilated atrium from the Fontan circuit with arrhythmia surgery and repair of any residual or recurrent lesions are now undertaken by some groups.[85,86]

The most common indication is refractory atrial arrhythmia with or without hemodynamic abnormalities.[87,88] The presence of severe systemic ventricular dysfunction not attributable to arrhythmia, elevated pulmonary vascular resistance or branch pulmonary artery hypoplasia contributing to raised pulmonary artery pressure may not benefit from Fontan conversion. Arrhythmia surgery involves epicardial electrophysiological studies intraoperatively, and cryoablation of lesions identified in right and occasionally in left atrium. Patients also have pacemaker implantation with a defibrillator if indicated for ventricular tachycardia. The advances in multisite pacing have also been applied to dyskinetic systemic ventricles in selected group.[89] With increasing experience, patients with significant atrioventricular valve regurgitation, and those with stenosis in the Fontan circuit that is deemed repairable have also been accepted for conversion.

Despite increasing experience with Fontan conversion, Mavroudis and colleagues found that 5.4% of their 111 patients required cardiac transplantation.[87] Hence, conversion surgery merely delays the inevitable.

### Transplantation in failing Fontan

When Fontan failure sets in, there is an inexorable hemodynamic and functional decline in the patients leading to death or cardiac transplantation. The early experience with transplantation in patients with Fontan circulation was of high operative mortality and morbidity.[90,91] The assumption that if a patient survives with a Fontan circulation, then the PVR is low enough for the right ventricle of the graft after cardiac transplantation was found to be incorrect in the early experience of Fontan transplants.

The considerations for cardiac transplantation are quite different in patients with failing Fontan circulation.[92] Anatomical substrates in isomerism and heterotaxy challenge the surgeon to achieve successful anastomosis of the graft in presence of abnormal situs.

Chronic cyanosis leads to polycythemia and coagulation abnormalities which are further complicated by hepatic dysfunction. Presence of PLE adds a metabolic and immunologic burden on an already decompensated circulation. Risk of bleeding during and after surgery is compounded by the presence of multiple acquired systemic to pulmonary arterial and systemic venous collateral vessels. The presence of panel reactive antibodies due to the use of allograft material also interfere with optimal immunosuppression.[93]

The substrate for pulmonary vascular dysfunction starts well before the Fontan circulation is established due to either abnormal pulmonary arteries, increased pulmonary blood flow for varying duration, pulmonary venous or left atrial obstruction, or chronic pulmonary thromboembolism.

Measurement of PVR in Fontan circulation is fraught with difficulties due to inability in accounting for collateral circulation, possibility of pulmonary arteriovenous malformation, low cardiac output state, presence of systemic venous obstruction, unequal distribution of flow to lungs, and possibility of pulmonary venous obstruction. All these factors multiply the error in accurate assessment of PVR.[93]

Pulmonary vascular disease was demonstrated in 10 patients who received a transplant late after a failing Fontan circulation (failure >1 year after Fontan completion), however, four patients who received a transplant within one year of their Fontan failure did not have pulmonary vascular disease.[94] Apart from the Fontan state that alters the flow dynamics in the pulmonary vascular bed, the Fontan preparation and duration of high pulmonary blood flow may also contribute to this finding.[32] Whether this elevated PVR documented early during follow-up of the transplant patients remains fixed or responds to pulmonary vasoactive agents and subsequently returns to baseline or normal values remains to be seen.

Cardiac transplantation is a definitive palliation in the Fontan journey. Despite the difficulties related to pretransplant assessment in failing Fontan circulation, the medium term outcome is reasonably good.

In a multi-institutional study in the United States, between 1993 and 2001, 70 patients had cardiac transplant for failing Fontan with a 5-year survival of 68%. Although the survival rates in postFontan patients as compared to other congenital heart disease were not significantly different, it remains slightly worse than patients with no congenital heart disease.[95,96] The long-term problems are no different than in any other indication for transplant including rejection. PVR may increase after transplantation due to better cardiac output through the ‘less than optimum’ pulmonary vascular bed. Early postoperative management should anticipate this consequence to reduce the morbidity.

## SUMMARY

The Fontan circulation offers the only definitive surgical palliation for patients with a wide variety of complex congenital heart diseases with a single dominant ventricle. It, however, sets a stage for hemodynamic and electrophysiological sequence of events that manifest when the circulation starts to fail. Improved understanding of patient selection, patient preparation, and surgical techniques has allowed us to reach a steady state in the early outcomes after achieving the Fontan circulation. The two new problems facing this group of patients are likely to be related to the late attrition of survivors and the growing population of high risk and borderline patients undergoing single ventricle palliation creating a heretofore unknown cohort that will challenge cardiologists, electrophysiologists, and surgeons, not to mention, the patients and their carers. Advances in management of heart failure have improved the late outcome Fontan circulation to a certain extent. Implantable ventricular assist devices may probably change the course of this circulation; however, until then this imperfect circulation would still be the only surgical option for this difficult patient population.
